# Microbial Nanocellulose Printed Circuit Boards for Medical Sensing

**DOI:** 10.3390/s20072047

**Published:** 2020-04-06

**Authors:** Jonathan D. Yuen, Lisa C. Shriver-Lake, Scott A. Walper, Daniel Zabetakis, Joyce C. Breger, David A. Stenger

**Affiliations:** Center for Bio-Molecular Science and Engineering, U.S. Naval Research Laboratory, Washington, DC 20375, USA; lisa.shriverlake@nrl.navy.mil (L.C.S.-L.); scott.walper@nrl.navy.mil (S.A.W.); daniel.zabetakis@nrl.navy.mil (D.Z.); Joyce.Breger@nrl.navy.mil (J.C.B.); david.stenger@nrl.navy.mil (D.A.S.)

**Keywords:** flexible electronics, nanocellulose, biosensing

## Abstract

We demonstrate the viability of using ultra-thin sheets of microbially grown nanocellulose to build functional medical sensors. Microbially grown nanocellulose is an interesting alternative to plastics, as it is hydrophilic, biocompatible, porous, and hydrogen bonding, thereby allowing the potential development of new application routes. Exploiting the distinguishing properties of this material enables us to develop solution-based processes to create nanocellulose printed circuit boards, allowing a variety of electronics to be mounted onto our nanocellulose. As proofs of concept, we have demonstrated applications in medical sensing such as heart rate monitoring and temperature sensing—potential applications fitting the wide-ranging paradigm of a future where the Internet of Things is dominant.

## 1. Introduction

In this report, we present our research on the viability of building functional medical sensors on ultra-thin sheets of microbially grown nanocellulose by forming printed circuit boards (PCBs) on them. Electronics built on very thin substrates of 20 microns and below are being extensively explored for various potential applications, particularly for sensors in medical applications [1,2,3,4,5]. Ultra-thin substrates are attractive because they are naturally conformal, self-adhering, ultra-lightweight, and translucent. Thus, they are ideal for applications which require imperceptibility [6,7,8], such as wear-and-forget health monitoring systems [1,2,4,5], electronic skin/noses [9,10,11,12,13,14], and bioelectronics tags and markers [15,16,17,18]. Different from typical “hard” electronics, which constitute objects-in-themselves, flexible electronics, particularly imperceptible electronics, can be envisioned as minimalized objects, or even non-objects, that can be seamlessly integrated or attached to other larger physical objects unobtrusively and without hindrance. Affixed on their targets, these flexible electronics constitute the Internet of Things (IoT), sharing and recording their perception of the surroundings and uploading the information to pertinent data centers, thereby increasing the connectivity of physical objects to the virtual network. Research and development of IoT are merely at its incipience [19,20,21]. The IoT global market value is projected to be around $7.1 trillion by 2030, with an estimated 30 billion IoT-related devices in operation globally [22]. While ultra-thin flexible electronics are poised to play an essential role in this new social and technological paradigm, technological problems exist that need to be addressed. For example, while ultra-thin polymer substrates are available, printing with typically hydrophilic inks on hydrophobic polymeric surfaces is challenging. Additionally, issues with breathability and biocompatibility hinder their utility for health-related applications, along with sustainability issues [23,24,25].

## 2. Materials and Methods

### 2.1. Materials and Components

All chemicals for nanocellulose sheet growth, such as sodium hydroxide (NaOH), sodium azide, D-(+)-Glucose, glycerol, and chitosan, were purchased from Fisher Scientific, Inc. (Pittsburgh, PA, USA). Glass wafers for plating of the sheets were purchased from University Wafer, Inc. (Boston, MA, USA). All surface mount electronic components were purchased from DigiKey (Thief River Falls, MN, USA). Chemicals for electroless plating were purchased from East Coast Electronic Material Supply LLC (Manchester, NH, USA). 

### 2.2. Nanocellulose Growth and Sheet Formation

*Gluconacetobacter xylinus* cultures were continuously maintained in HS medium 1 as static cultures at 30 °C. Mother cultures were maintained in 50 mL conical tubes containing 5 mL of sterile medium. These cultures were left undisturbed for 7 days to allow the culture to develop. To generate the inoculum, the pellicle was dislodged, and cells dispersed using a vortexer. The culture was vortexed at maximum speed ten times for 3 s. During the vortexing process, the pellicle and any diffuse cellulose material precipitated to the bottom third of the conical tube. Using a sterile pipette, 1 mL of suspended bacteria were transferred to fresh 50 mL HS medium in sterile 100 mm crystallization dishes that had previously been covered with aluminum foil and autoclaved. The inoculated crystallization dishes were covered again with the original aluminum foil and transferred to the incubator, where they were incubated undisturbed for seven days. Following this incubation, 50 mL of fresh HS medium was gently added to the surface of the basal pellicle. The cultures were returned to the incubator for one week to allow a second pellicle to form. The “feeding” process was repeated at one-week intervals for a total of five weeks to form four uniform pellicles plus the initial basal pellicle.

Harvested nanocellulose pellicles were incubated at 90 °C in 0.5 M NaOH for 1 h to denature contaminating proteins and bacteria. The pellicles were then transferred to 13 × 9 in Pyrex vessels, where they were soaked extensively in water over a 48-h period. Water washes were continued until pH measurements indicated the removal of base materials. Pellicles were stored in water containing 0.02% sodium azide to inhibit microbial growth. To form the sheets, pellicles were spread across 10 mm glass wafers and manually smoothened to minimize air bubbles trapped between pellicle and wafer. Overnight, the millimeter-thick pellicles dry down to microns-thick nanocellulose sheets. The sheets were found to be thermally stable up to 200 °C with 30 min of annealing. As reported in literature [26], bacterial nanocellulose has thermal conductivities of 0.37 Wm^−1^K^−1^ along the sheet thickness and 1.29 Wm^−1^K^−1^ in-plane, and a thermal expansion coefficient of 3 pmm∙K^−1^.

### 2.3. AFM Characterization

Atomic Force Microscopy was performed to characterize the morphology of the nanocellulose sheet. A Digital Instruments Dimension 3100 AFM was used to characterize the nanocellulose samples, using triangular Si_3_N_4_ microcantilevers (Nanoprobe, Bruker AFM Probes Americas, Camarillo, CA, USA).

### 2.4. Thickness Measurements

Thickness measurements of the nanocellulose sheets were performed on a KLA-Tencor Alpha-Step D-500 profilometer, on which selected areas on each wafer were scratched with a tweezer to reveal the underlying wafer from which the profilometry measurement was performed. Profilometer measurements of 5 samples over multiple locations on each sample resulted in an average of 2.94 μm thickness with a standard deviation of 0.47 μm.

### 2.5. Electroless Plating on Nanocellulose Sheets

Inkjet printing of Pd catalyst ink patterns was performed with a FujiFilm Dimatix DMP-2831 Materials Printer. Cataposit 44 (Rohm & Haas, East Coast Electronic Material Supply LLC, Manchester, NH, USA), used as received, was diluted 1:6 with 11% hydrochloric acid and deionized water, and filtered into a DMC-11610 cartridge (10 pL drop-size) with a 0.2 µm Nalgene PTFE syringe filter. During printing, the platen temperature was set at 37 °C, and the cartridge temperature was left at room temperature. Printing was performed at a resolution of 1270 DPI (corresponding to dot pitch of 20 μm), with the jetting voltage range between 15–35 V and with only 4 of the 16 jets used. After printing, the substrate was immersed in DI water to remove the acid in the ink, and the printed nanocellulose sheet was then peeled off the glass wafer it was mounted on. The peeled sheet was remounted while still in DI water onto a transparency sheet, then removed from the DI bath and air-dried. Upon drying, a double-sided tape was attached to the edges of the transparency to secure the nanocellulose sheet. 

Three layers of different metals, copper, nickel, and gold, were plated onto the catalyst patterns by immersing the mounted transparency sheet into specific chemical baths. Plating of copper was carried out using Cuposit 328 electroless copper plating solution at 55–57 °C; plating of nickel was carried out using Duraposit SMT88 electroless nickel plating solution at 88 °C; and gold, with Aurolectroless 520 gold plating solution at 88 °C. Upon the completion of each plating step, the samples were soaked in water (three times) to remove the residual electroless bath. After all the plating steps, the samples were left overnight to air-dry. 

### 2.6. Soldering of Surface Mount Electronics and Electrodes on Nanocellulose

Field’s Metal (Roto144F, Rotometals, San Leandro, CA, USA), a low melting point, low toxicity metal alloy [27,28,29,30,31] consisting of bismuth, tin, and indium, was used as the solder. To connect the surface mount devices to the plated electrodes on the nanocellulose sheet, a solder flux was first applied to the metal surface to ensure better wetting of the solder. Field’s Metal was then applied to the treated metal surface. After applying Field’s Metal to selected areas of the plated metal layer, the glass wafer with the nanocellulose sheet was placed on a hot plate at 75 °C–80 °C, allowing the solder to re-melt. The surface-mount electronics were then placed on the melting solder pads. After placement, the glass wafer was taken off the hot plate and allowed to cool, whereupon the solder solidified, and the electronics were welded firmly onto the sheet. The assembled nanocellulose PCB was then tested for electrical continuity. 

### 2.7. Electrical Testing 

The pulse oximeter was tested with an Agilent 4155B in voltage versus time mode at an applied voltage of 5 V over a period of 2 min per measurement. Pulses were calculated using an Excel macro, which counted the number of times a value was crossed as the reading oscillates over a 60s period, then divided in half. The temperature sensor was connected to a test board (MAX30205EVSYS) provided by Maxim Integrated, Inc. (San Jose, CA, USA) through a ribbon cable. The test board was, in turn, connected to a PC via a USB connection. The software was provided by Maxim Integrated, Inc., which captured temperature data generated by the temperature sensor.

### 2.8. Notes

We would like to point out that the manufacture of the nanocellulose sheets and certain aspects of the printed circuit board fabrication process are similar or the same to methods described in our previous publications [32,33].

## 3. Results and Discussion

### 3.1. Synthesis of Ultra-Thin Nanocellulose Sheets

**Figure 1:**
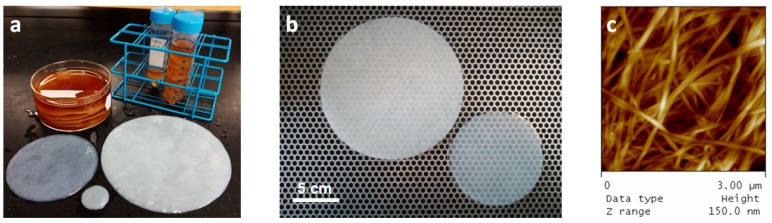
(**a**) Nanocellulose pellicles shown as grown in media and after cleaning. Multiple pellicles can be grown in one vat, and pellicles of various sizes can be grown. Pellicle sizes are 1 in, 4 in, and 6 in. (**b**) Nanocellulose pellicles dried into sheets on glass wafers, on the left is a 6 in sheet, and on the right is a 4 in a sheet. (**c**) AFM height measurements of a nanocellulose sheet.

In response to the issues with plastic substrates, as mentioned at the end of the introduction, we have recently reported a process to create microbial nanocellulose sheets thinner than 20 μm, which represent a new material class. Our research thus far has shown microbial nanocellulose to have many qualities superior to existing plastic as substrates for flexible electronics, especially for health-related applications [32,33]. Unlike plastics, microbial nanocellulose is highly chemical and solvent resistant, mechanically strong, [34,35,36,37] water permeable, and biocompatible [35,38,39]—a unique set of characteristics which we took advantage of to address the above-mention problems with plastic substrates. Nanocellulose pellicles are grown through a fermentative process at 30 °C, mediated by a bacterial agent *Gluconacetobacter xylinus*. At the air-water interface of a media bath, *Gluconacetobacter xylinus* consumes simple sugars (such as glucose) or polyols (such as glycerol), and excretes long, uniform cellulose chains as a by-product. The cellulose chains aggregate to form fibers of high crystallinity of close to 90%, which, in turn, agglomerate to form millimeter-thick cellulose gel pellicles at the air-water interface. Our nanocellulose sheets are grown in-situ from microbial broth as millimeter-thick gel layers and can be of any arbitrary size or shape as determined by the growth vat, as shown in Figure 1a. Multiple pellicles can be grown in one growth vat by filling the growth vat with fresh media, creating a new air-water interface above the growth location. The bacteria will rise to the new surface and begin the growth of a new pellicle there, while the original pellicle remains stuck and submerged in the original location, also shown in Figure 1a. After the pellicles are cleaned to remove the bacteria and media, they can then be laminated onto any dry surface to dry into microns-thick sheets. Moistening the film does not return it to the gel state; rather, it retains its sheet-like characteristics. There has been much research and many industrial initiatives to commercialize bacterial nanocellulose [40]. In fact, the process of manufacturing this unique material on an industrial scale at relatively low cost has precedence in the manufacture of nata de coco, a lower quality microbially grown, edible nanocellulose that is common in Asian food products [41,42]. Furthermore, bacterial cellulose is now currently manufactured in large quantities in the cosmetics industry under the commercial name of *biocellulose* for hydrating face masks, with potential medical applications as wound/burn dressings [43,44,45,46]. To date, few reports exist describing the direct use of microbially grown sheets of nanocellulose for the manufacturing of flexible electronics. Most applications typically rely on the maceration of the pellicle and the subsequent incorporation of the homogenized nanocellulose fibers into a cast film or composite. We show, however, that there are numerous benefits in utilizing the original pellicle structure, as we are able to tune its properties. Another interesting aspect of our sheets is that they adhere very strongly onto inorganic surfaces such as a glass wafer, as shown in Figure 1b, an aspect which we will describe later. This has allowed us to easily develop fabrication processes on our nanocellulose sheet without fear of distortion or delamination.

### 3.2. Nanocellulose Characteristics for Flexible Electronics 

**Figure A1:**
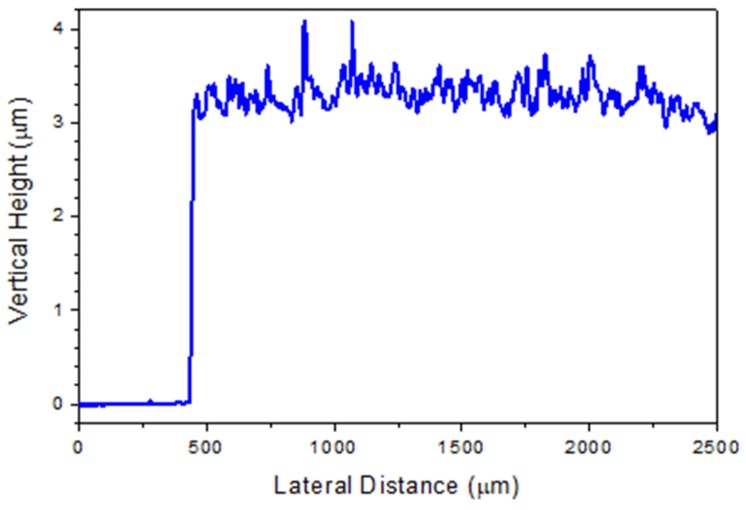
Profilometer profile of a typical nanocellulose sheet.

Atomic Force Microscopy was performed on the nanocellulose sheets, depicted in Figure 1c, revealing a porous network of nanocellulose fibers between 30 nm to 100 nm in thickness, with some fibers bundled to form thicker cables. The thickness of the sheets was determined to range from 0.5 µm to 20 µm per profilometry measurements and was dependent on the length of feeding. The sheets used in our experiments were around 3 µm, as measured in Figure A1 in the Appendix A. However, in spite of their thinness, microbial nanocellulose sheets are highly chemical and solvent resistant and mechanically strong as well. The porosity of our nanocellulose sheets makes them amenable to the wicking effect, allowing the absorption of most liquids into the nanocellulose matrix. With the exception of our material, flexible, free-standing substrates below 20 μm that contain a porous network are extremely rare and very difficult to manufacture in bulk. The porosity of our cellulose sheet allows it to be amenable to printing processes. It enables the infusion of the palladium catalyst ink that is used as a precursor to the electroless plating of metals onto the nanocellulose sheet during the fabrication of our nanocellulose PCB.

Another advantage of our ultra-thin microbial nanocellulose sheets is their self-adhering nature. Dry nanocellulose can be laminated onto many inorganic surfaces, such as metal or glass, simply by moistening the sheet, laying it on the desired surface, and allowing it to dry. A strong adhesive bond can be formed between nanocellulose sheets and these surfaces, where mechanical force, such as scratching, or physical modification of the sheet, such as wetting, will result in the removal of the sheet. In fact, scratching a dried nanocellulose on a glass wafer will only result in selective tearing of the sheet at the location where the scratch was made. The strong bond between nanocellulose and these inorganic surfaces is believed to originate from hydrogen bonds from the hydroxyl-terminated groups that decorate the sides of the cellulose polymer chains [47]. Adhesion tape tests have been performed on Kapton, glass, and aluminum, with videos and a short description available in the Supplementary Materials. As mentioned previously, this characteristic has allowed us to easily develop fabrication processes on nanocellulose sheets without worrying about distortion or delamination [32,33]. Being ultrathin and flexible, our nanocellulose sheets and nanocellulose electronics conform onto many surfaces and remain attached to the location. The added advantage of microbial nanocellulose being biocompatible means that flexible medical-related electronics can be directly attached to the skin without the need for any adhesive layer. Indeed, we have found that nanocellulose sheets can remain attached to human or chicken skin for over 12 h if not inordinately disturbed [32].

The above-mentioned characteristics of nanocellulose sheets present opportunities for various applications, especially for flexible electronics. To date, we have developed various processing capabilities for the fabrication of electronics on our nanocellulose sheets. The sheets are amenable to various microfabrication processes, of which we have demonstrated optical lithography, vacuum evaporation, and dry etching [48]. Given the advantage of the low-cost manufacturability of our nanocellulose sheets, relative to the microfabrication processes described previously, we want to further this inherent advantage by incorporating lower cost, solution-based electronic fabrication processes, namely conventional printing and plating. With their porosity and hydrophilicity, nanocellulose sheets easily wick inks and are, therefore, amenable to printing-based processes. So far, we have demonstrated the ability to print with a Dimatix Materials Printer, both insulating and semiconducting materials on our nanocellulose sheets, including an insulating polymer, SU8, and a semiconducting polymer, PEDOT:PSS. Additionally, the ability of nanocellulose to soak up liquids also enable plating chemicals to penetrate the sheets and allow metallic plating reactions to occur within their matrix. In our case, to create metallic wires on nanocellulose sheets, we have exploited electroless plating, that is, catalyst-mediated, spontaneous reduction of metals from solution onto a pre-treated surface. By combining the printing of a plating catalyst ink together with electroless plating, we are able to create metallic wiring traces on our nanocellulose sheet, which constitute the basis of a printed circuit board. We have also developed a low-temperature soldering process to allow the attachment of surface mount components onto the nanocellulose sheet, thus creating a nanocellulose-based PCB.

### 3.3. Nanocellulose PCB Fabrication

**Figure 2:**
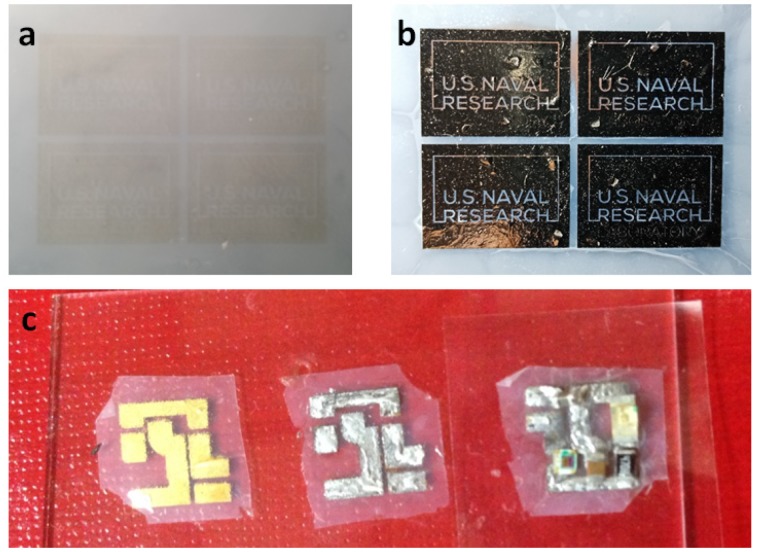
(**a**) Ink-jet printed NRL (U.S. Naval Research Laboratory) logo on nanocellulose with a palladium-based catalyst ink. (**b**) Electroless copper-then-nickel-plated NRL logo on nanocellulose, based on the ink pattern in (**a**). (**c**) Soldering process on a nanocellulose printed circuit board (PCB). Left: A nanocellulose sheet with an electroless-plated wiring pattern of gold-plated, nickel-plated copper. Middle: A similar pattern with Field’s Metal soldered onto the surface. Right: Electronic components mounted onto the soldered wiring pattern.

To fabricate the nanocellulose PCB, a diluted version of Cataposit 44 was used for printing as higher concentrations would induce the solidification of plating too rapidly, resulting in distorted and rough metal films. Using our Dimatix Materials Printer, we first inkjet-printed an ink print pattern onto a nanocellulose sheet laminated onto a glass wafer, as shown in Figure 2a. The sheet was then rinsed in DI water to remove excess acid in the ink, which might damage the nanocellulose. The sheet was then dried before being immersed in a copper plating bath to form a plated copper base layer, which would serve as a subsequent plating layer for a thin layer of electroless-plated nickel. The copper bath used in the process has to be strictly controlled at a plating temperature range between 55 °C to 57 °C, again to prevent the plating reaction from occurring too rapidly. Such rigorous temperature control was observed for all subsequent plating procedures to regulate the speed of the plating reaction. The resultant nickel-plated copper metallic wiring pattern based on the ink print (which can only be faintly discerned in Figure 2a due to the dilute nature of the ink), is shown in Figure 2b. A comparison of the two images shows good fidelity between them. We note that a relatively smooth film can be formed directly on top of the nanocellulose sheet despite the high porosity and roughness of the nanocellulose surface. The plated sheet was then rinsed thrice to remove any residual plating solution and left to dry overnight before soldering is performed on it. For our PCBs, we decided to plate an additional layer of gold to allow us to have a chemically inert metal surface for subsequent soldering, as shown in the left of Figure 2c.

To enable us to attach surface mount components onto the metallic wiring patterns on the nanocellulose surface, we have developed a low-temperature soldering process utilizing a low melting-point, low toxicity, bismuth and indium-based alloy known commonly as Field’s Metal which has a melting point of around 60 °C. Since our devices are designed for medical applications, we wanted a lead-free soldering process, and Field’s metal fulfilled that requirement with the added benefit of easy handling due to its low melting point. Surprisingly, normal soldering techniques are amenable for the soldering of Field’s Metal. First, flux was brushed onto a section of the metallic wiring, where the solder would subsequently be applied to clean the metal surface. After application of flux, melted solder, applied with a soldering iron, wicked readily onto the cleaned area, forming a strong weld. This procedure was repeated all over the metallic wiring pattern where surface mount components were to be placed, as shown in the middle of Figure 2c. To mount the electronic components, the glass wafer with the nanocellulose sheet was placed on a hot plate set at 75 °C to re-melt the solder, and the surface mount components were then carefully laid on the soldered areas. Upon completion of the process, the wafer was removed from the hot plate and allowed to cool for the solder to resolidify. The final PCB, also shown in the right of Figure 2c, is our first iteration of a nanocellulose PCB heart rate monitor.

Our successful fabrication of a nanocellulose PCBs is a rare demonstration of the electroless plating of metallic wires to create flexible electronics. It is also the first demonstration of plating on a flexible porous surface. Electroless plating on flexible surfaces is an under explored process due to the difficulty of printing the aqueous, acidic palladium nanoparticle catalyst ink onto the usual hydrophobic plastic substrates. Our approach differs from current approaches to fabricating standard flexible PCBs, which typically involve laminating a copper sheet, several tens of microns thick, onto a plastic sheet, also several tens of microns thick. Copper from the copper sheet is then etched onto the plastic sheet to form the desired pattern. This results in flexible electronics that are very thick and tend to lack the flexibility of thinner sheets. To create thinner flexible electronics, the current most popular approach to forming metallic wires on flexible surfaces is to use commercially available formulated silver nanoparticle inks or silver precursor inks to deposit patterns of silver. However, there are limitations with polymer substrates since most polymers cannot withstand the high temperature anneal required to achieve high conductivities, and that this printing process is currently limited to silver.

In comparison, creating PCBs on semi-porous substrates such as nanocellulose offers a number of advantages over existing technologies mentioned above. Firstly, using our process, we can use many more metals for plating, for example, copper, nickel, silver, gold, palladium, and platinum. Secondly, not only can the hydrophilic and porous nanocellulose wick biological analytes to the decal on the other side of the substrate for analysis, it can also serve as a storage and transport tool to retain and selectively deliver analytes to the sensing electronics of interest. Thirdly, we can exploit the porosity of the nanocellulose sheet to create metallic vias-metallic wires at selected areas of the nanocellulose sheet that can enable electric connections between electronic infrastructures on opposite sides of the nanocellulose sheet. Our techniques are, therefore, simpler than the standard PCB process, in which through-holes have to be punched into the sheet to enable such vertical connections through the sheet.

### 3.4. Examples of Medical Application of Nanocellulose PCBs

**Figure 3:**
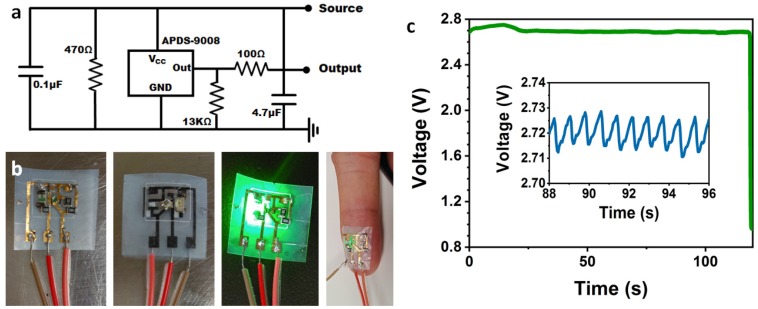
(**a**) Circuit diagram of the heart rate monitor. (**b**) Front (left) and back (middle-left) of the completed heart rate monitor, the device in operation (middle-right), and the device in operation on a finger. (**c**) The green line on the outset graph shows the heart rate measurement of the test subject where the monitor was removed at around t = 119 s, indicating a stable measurement over a lengthy period of time, and clear cessation of operation upon loss of contact to the subject. The inset graph with the blue line shows a magnified view of measurements over a narrow period of time for clarity, with the voltage oscillations corresponding to palpitations.

Using the nanocellulose PCB fabrication process described above, we have developed a few examples of nanocellulose PCBs capable of medical sensing applications. Figure 3a shows the circuit layout of a nanocellulose reflective pulse monitor, consisting of a green LED with peak intensity at 515 nm and a luminosity of 3000 mcd (AM2520ZGC09, Kingbright, Taipei, Taiwan), coupled with a phototransistor (APDS-9008, Avago, peak sensitivity at 565 nm). As the heart beats, the ebb and flow of blood to the section of the body to which our pulse monitor is attached causes cyclical variations in the absorption of light from the LED. As more blood is pumped in, more absorption of light occurs. This results in an inverse, but an equivalently cyclical change in reflectance intensity which can be captured by the phototransistor. The capacitor and resistors in the PCB constitute a low bandpass filter, which is attached to the pulse monitor to help eliminate the 60Hz noise from AC power in the environment. The physical embodiment of the circuit, in the form of a decal built on our nanocellulose PCB, is shown (back and front) in Figure 3b, with the soldered circuit components linked to each other via copper-nickel-gold wires plated into the nanocellulose sheet. We exploited the porosity of the nanocellulose to plate vias by allowing the palladium catalyst ink to completely soak through the nanocellulose sheet. With a relatively thick nanocellulose sheet, we can control the penetration depth of the catalyst ink simply by varying the ink concentration and printing volume, thereby printing vias selectively on our nanocellulose sheet. Using the vias, we have placed only the essential electronic components needed for heart rate monitoring (the LED and the phototransistor) on one side and the other components (capacitors and resistors) on the other side. The purpose of doing so is to minimize the extent of the test subject’s skin coming into contact with electronic components, thereby protecting the user from potential contamination from the electronics. The image to the middle-right right-most of Figure 3b shows the front side of the device in operation with the LED-lit from behind.

To measure the heartbeat of a test subject, the nanocellulose component of the reflective pulse monitor lightly moistened and laminated onto the finger of the test subject. Upon drying, the nanocellulose sheet creates a strong weld on the skin of the subject, due to the presence of hydrogen bonds on the cellulose polymer chain, sealing the electronic components to the skin surface. This sealing allows intimate contact of the LED and phototransistor with the skin, enhancing measurement accuracy. Flexible wires have been soldered onto bus-leads, included on the circuit layout, allowing us to directly connect the nanocellulose PCB to an electronic measurement setup. To monitor the outgoing electronic signals from the nanocellulose reflective pulse monitor, we used an Agilent 4533 Parametric Analyzer, set at voltage versus time mode, to sample the cyclical voltage variations produced by the phototransistor as the test subject’s heartbeat is tracked over the course of a few minutes. In Figure 3c, we present, for the sake of clarity, only a small subset of the total data obtained with our pulse monitor in operation. The relatively regularly-spaced pulses in voltage can be clearly defined, such that an average heart rate of 72 bpm of the test subject can be determined.

**Figure 4:**
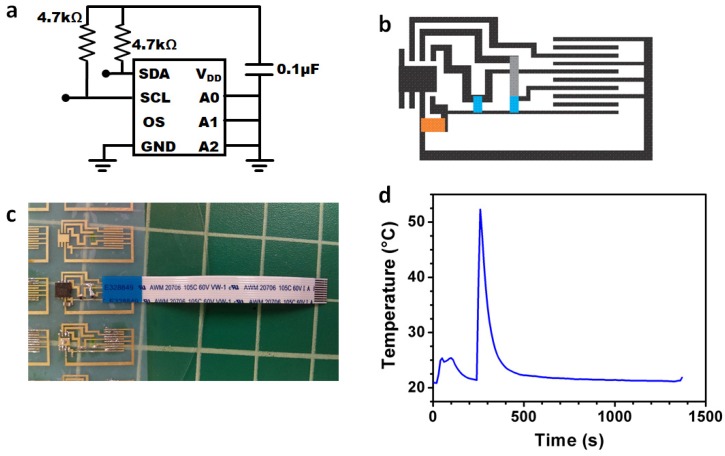
(**a**) Circuit diagram of the temperature sensing device. (**b**) Circuit board layout of the temperature sensing device with blue as the resistors, orange as the capacitor, and grey as the bridging connection. (**c**) Completed temperature sensor on nanocellulose. (**d**) Temperature output of the sensor tested over a period of time, first increase in temperature was due to contact with a finger touching the device, and the second increase was when the device was placed on the hotplate, and the hotplate turned off and left to cool.

In another incarnation of a nanocellulose PCB medical sensing device, we tested the capability of our nanocellulose PCB technology to support a more complex circuit layout with the inclusion of an integrated circuit surface mount device and its attendant surface-mount components. In doing so, we created a design for a nanocellulose PCB temperature sensing device that implements a Maxim Integrated Human Body Temperature Sensor (Maxim Integrated, MAX30205)—a low-voltage, low-current, I2C-compatible temperature sensor cased in a QFN-type (Quad Flat No-leads) surface mount package. The I2C interface provided by Maxim Integrated has a USB port that allows us to interrogate the temperature sensor by directly connecting it to a PC with installed software also provided by Maxim Integrated. The temperature sensor decal can simply be connected to the I2C interface via a ribbon cable connector. The requirements layout for the MAX30205 to connect with the I2C interface is shown in Figure 4a, along with a design of the circuit layout in Figure 4b. We note that due to the spatial limitations imposed by the circuit design and the need for the outputs to be routed to the ribbon connector bus, a bridging wire connection is needed above the main circuit layout. This bridging connection, which cannot short with the main circuit layout, is shown by a grey, semi-transparent connection in Figure 4b.

To create the bridging connection, we exploited the thinness and flexibility of the nanocellulose sheet to connect a plated nanocellulose “wire” between the two nodes. During the fabrication of the main circuit wiring pattern, a second wiring pattern was created and was then laser-cut and peeled away from the main sheet. The wire was then mounted onto the nodes at the same time when the rest of the surface mount components were soldered, creating the bridging connection, as shown in Figure 4c. Figure 4d shows the device in operation, showing a graph of the temperature responses of the sensor with time. The first temperature spike at 22 s occurred when the decal is placed on the test subject’s hand, and the second temperature spike at 244 s occurred when the decal is placed onto a hotplate, which was then turned off and allowed to cool. We note that all the devices were hand soldered, but with better soldering tools, more complex architectures can potentially be achieved.

## 4. Conclusions

In conclusion, we have demonstrated the viability of a novel material, ultra-thin microbially grown nanocellulose sheets, to be used to create flexible, printed electronic circuit boards. Our nanocellulose PCBs can be cheaply manufactured from the bottom-up using ambient, solution-based processing techniques, such as microbial vat-growth, inkjet printing, and electroless plating. The processing techniques developed, while novel, nonetheless can be easily integrated into current manufacturing processes. While we have demonstrated applications in medical sensing, such as heart rate monitoring and temperature sensing, this is merely a proof-of-concept. We can envision applications as varied as health care, environmental sensing, smart home, and smart retail, all of which fit into the wide-ranging paradigm of a future where the Internet of Things is dominant. In the future, we hope to expand our nanocellulose PCBs to further develop more interesting electronic devices and exploit the flexibility of our PCBs to create more novel applications towards this new paradigm.

## Supplementary Materials

The following are available online at https://www.mdpi.com/1424-8220/20/7/2047/s1: three videos showing tape tests on of nanocellulose laminated on three different surfaces—kapton, glass and aluminum.

## Appendix B. Adhesion Tape Tests Experimental and Video Description

Rectangular samples of nanocellulose sheets were cut from a larger sheet attached to a glass wafer, moistened, peeled, and laminated wet onto three surfaces—a sheet of Kapton, a glass wafer and an aluminum plate, and allowed to dry. Scotch tape was then tightly adhered to each of the surfaces, covering parts of the surface and a section of the nanocellulose sheet, and then the tape was peeled off from each surface. For the aluminum plate, vigorous rubbing with a pressed finger was also performed on the nanocellulose sheet prior to peeling off the tape. As shown in the videos, we observed that the nanocellulose sheets remained adhered to the Kapton sheet and the glass wafer after the tape peeling, while the nanocellulose sheet on the steel plate cannot be removed by rubbing, but came off easily with the tape when it was peeled off.

## References

[1] Khan Y., Ostfeld A.E., Lochner C.M., Pierre A., Arias A.C. (2016). Monitoring of Vital Signs with Flexible and Wearable Medical Devices. Adv. Mater..

[2] Liu Y., Pharr M., Salvatore G.A. (2017). Lab-on-Skin: A Review of Flexible and Stretchable Electronics for Wearable Health Monitoring. ACS Nano.

[3] Ling H., Liu S., Zheng Z., Yan F. (2018). Organic Flexible Electronics. Small Methods.

[4] Gao W., Ota H., Kiriya D., Takei K., Javey A. (2019). Flexible Electronics toward Wearable Sensing. Acc. Chem. Res..

[5] Yang Y., Gao W. (2019). Wearable and flexible electronics for continuous molecular monitoring. Chem. Soc. Rev..

[6] Kaltenbrunner M., White M.S., Głowacki E.D., Sekitani T., Someya T., Sariciftci N.S., Bauer S. (2012). Ultrathin and lightweight organic solar cells with high flexibility. Nat. Commun..

[7] Kaltenbrunner M., Sekitani T., Reeder J., Yokota T., Kuribara K., Tokuhara T., Drack M., Schwödiauer R., Graz I., Bauer-Gogonea S. (2013). An ultra-lightweight design for imperceptible plastic electronics. Nature.

[8] Someya T., Bauer S., Kaltenbrunner M. (2017). Imperceptible organic electronics. MRS Bull..

[9] Wang X., Dong L., Zhang H., Yu R., Pan C., Wang Z.L. (2015). Recent Progress in Electronic Skin. Adv. Sci..

[10] Biji K.B., Ravishankar C.N., Mohan C.O., Srinivasa Gopal T.K. (2015). Smart packaging systems for food applications: A review. J. Food Sci. Technol..

[11] Son M., Park T.H. (2018). The bioelectronic nose and tongue using olfactory and taste receptors: Analytical tools for food quality and safety assessment. Biotechnol. Adv..

[12] Takei K., Gao W., Wang C., Javey A. (2019). Physical and Chemical Sensing With Electronic Skin. Proc. IEEE.

[13] Yang J.C., Mun J., Kwon S.Y., Park S., Bao Z., Park S. (2019). Electronic Skin: Recent Progress and Future Prospects for Skin-Attachable Devices for Health Monitoring, Robotics, and Prosthetics. Adv. Mater..

[14] Wang J., Yu J., Wang T., Li C., Wei Y., Deng X., Chen X. (2020). Emerging Intraoral Biosensors. J. Mater. Chem. B.

[15] Magliulo M., Mulla M.Y., Singh M., Macchia E., Tiwari A., Torsi L., Manoli K. (2015). Printable and flexible electronics: From TFTs to bioelectronic devices. J. Mater. Chem. C.

[16] Someya T., Bao Z., Malliaras G.G. (2016). The rise of plastic bioelectronics. Nature.

[17] Inal S., Rivnay J., Suiu A.-O., Malliaras G.G., McCulloch I. (2018). Conjugated Polymers in Bioelectronics. Acc. Chem. Res..

[18] Yu Y., Nyein H.Y.Y., Gao W., Javey A. (2019). Flexible Electrochemical Bioelectronics: The Rise of In Situ Bioanalysis. Adv. Mater..

[19] Gershenfeld N., Krikorian R., Cohen D. (2004). The Internet of Things. Sci. Am..

[20] Atzori L., Iera A., Morabito G. (2010). The Internet of Things: A survey. Comput. Netw..

[21] Gubbi J., Buyya R., Marusic S., Palaniswami M. (2013). Internet of Things (IoT): A vision, architectural elements, and future directions. Future Gener. Comput. Syst..

[22] Hsu C.-L., Lin J.C.-C. (2016). An empirical examination of consumer adoption of Internet of Things services: Network externalities and concern for information privacy perspectives. Comput. Hum. Behav..

[23] Halden R.U. (2010). Plastics and Health Risks. Annu. Rev. Public Health.

[24] Köhler A.R. (2013). Challenges for eco-design of emerging technologies: The case of electronic textiles. Mater. Des..

[25] Ceballos D.M., Dong Z. (2016). The formal electronic recycling industry: Challenges and opportunities in occupational and environmental health research. Environ. Int..

[26] Kargarzadeh H., Ahmad I., Thomas S., Dufresne A. (2017). Handbook of Nanocellulose and Cellulose Nanocomposites.

[27] Ainla A., Xu S., Sanchez N., Jeffries G.D.M., Jesorka A. (2012). Single-cell electroporation using a multifunctional pipette. Lab Chip.

[28] Paglia D.N., Wey A., Hreha J., Park A.G., Cunningham C., Uko L., Benevenia J., O’Connor J.P., Lin S.S. (2014). Local vanadium release from a calcium sulfate carrier accelerates fracture healing. J. Orthop. Res..

[29] Khan W., Jia Y., Madi F., Weber A., Ghovanloo M., Li W. A Miniaturized, Wirelessly-Powered, Reflector-Coupled Single Channel Opto Neurostimulator. Proceedings of the 2018 IEEE Micro Electro Mechanical Systems (MEMS).

[30] Panwar J., Roy R. (2019). Integrated Field’s metal microelectrodes based microfluidic impedance cytometry for cell-in-droplet quantification. Microelectron. Eng..

[31] Naskar S., Kumaran V., Markandeya Y.S., Mehta B., Basu B. (2020). Neurogenesis-on-Chip: Electric field modulated transdifferentiation of human mesenchymal stem cell and mouse muscle precursor cell coculture. Biomaterials.

[32] Yuen J.D., Walper S.A., Melde B.J., Daniele M.A., Stenger D.A. (2017). Electrolyte-Sensing Transistor Decals Enabled by Ultrathin Microbial Nanocellulose. Sci. Rep..

[33] Yuen J.D., Baingane A., Hasan Q., Shriver-Lake L.C., Walper S.A., Zabetakis D., Breger J.C., Stenger D.A., Slaughter G. (2019). A Fully-Flexible Solution-Processed Autonomous Glucose Indicator. Sci. Rep..

[34] Yamanaka S., Watanabe K., Kitamura N., Iguchi M., Mitsuhashi S., Nishi Y., Uryu M. (1989). The structure and mechanical properties of sheets prepared from bacterial cellulose. J. Mater. Sci..

[35] Gatenholm P., Klemm D. (2010). Bacterial Nanocellulose as a Renewable Material for Biomedical Applications. MRS Bull..

[36] Gea S., Bilotti E., Reynolds C.T., Soykeabkeaw N., Peijs T. (2010). Bacterial cellulose–poly(vinyl alcohol) nanocomposites prepared by an in-situ process. Mater. Lett..

[37] Iguchi M., Yamanaka S., Budhiono A. (2000). Bacterial cellulose—A masterpiece of nature’s arts. J. Mater. Sci..

[38] Gromet Z., Schramm M., Hestrin S. (1957). Synthesis of cellulose by Acetobacter xylinum. 4. Enzyme systems present in a crude extract of glucose-grown cells. Biochem. J..

[39] Klemm D., Schumann D., Kramer F., Heßler N., Hornung M., Schmauder H.P., Marsch S. (2006). Nanocelluloses As Innovative Polymers in Research and Application.

[40] Gama M., Dourado F., Bielecki S. (2016). Bacterial Nanocellulose: From Biotechnology to Bio-Economy.

[41] Shi Z., Zhang Y., Phillips G.O., Yang G. (2014). Utilization of bacterial cellulose in food. Food Hydrocoll..

[42] Ullah H., Santos H.A., Khan T. (2016). Applications of bacterial cellulose in food, cosmetics and drug delivery. Cellulose.

[43] Petersen N., Gatenholm P. (2011). Bacterial cellulose-based materials and medical devices: Current state and perspectives. Appl. Microbiol. Biotechnol..

[44] Mohite B.V., Patil S.V. (2014). A novel biomaterial: Bacterial cellulose and its new era applications: BC and Its New Era Applications. Biotechnol. Appl. Biochem..

[45] Lee C.K., Hsu K.C., Cho J.C., Kim Y.J., Han S.H., Amorepacific Corp (2016). Method for Manufacturing a Cosmetic Bio-Cellulose Mask Pack Sheet and Use Thereof. U.S. Patent.

[46] Morganti P., Morganti G., Chen H.D., Gagliardini A. (2019). Beauty Mask: Market and Environment. J. Clin. Cosmet. Derm..

[47] Gardner D.J., Oporto G.S., Mills R., Samir M.A.S.A. (2008). Adhesion and Surface Issues in Cellulose and Nanocellulose. J. Adhes. Sci. Technol..

[48] Daniele M.A., Yuen J.D. (2017). Pattern Definition of Nanocellulose Sheets Through Selective Ashing via Lithographic Masking. U.S. Patent.

